# Effectiveness of core needle biopsy in the diagnosis of thyroid lymphoma and anaplastic thyroid carcinoma: A systematic review and meta-analysis

**DOI:** 10.3389/fendo.2022.971249

**Published:** 2022-09-20

**Authors:** Vincent Vander Poorten, Nathan Goedseels, Asterios Triantafyllou, Alvaro Sanabria, Paul M. Clement, Oded Cohen, Pawel Golusinski, Orlando Guntinas-Lichius, Cesare Piazza, Gregory W. Randolph, Alessandra Rinaldo, Ohad Ronen, Maria E. Cabanillas, Ashok R. Shaha, Yong Teng, Ralph P. Tufano, Michelle D. Williams, Mark Zafereo, Alfio Ferlito

**Affiliations:** ^1^ Otorhinolaryngology, Head and Neck Surgery, University Hospitals Leuven, Leuven, Belgium; ^2^ Department of Oncology, Section Head and Neck Oncology, KU Leuven, Leuven, Belgium; ^3^ Department of Pathology, Liverpool Clinical Laboratories and School of Dentistry, University of Liverpool, Liverpool, United Kingdom; ^4^ Department of Surgery, School of Medicine, Universidad de Antioquia-Ips Universitaria, Medellín, Colombia; ^5^ Department of Oncology, Section General Medical Oncology, KU Leuven, Leuven, Belgium; ^6^ Department of Otolaryngology, Head and Neck Surgery, Soroka Medical Center, affiliated with Ben Gurion University of the Negev, Be’er Sheva, Israel; ^7^ Department of Otolaryngology and Maxillofacial Surgery, University of Zielona Gora, Zielona Góra, Poland; ^8^ Department of Otorhinolaryngology, Institute of Phoniatry/Pedaudiology, Jena University Hospital, Jena, Germany; ^9^ Department of Otorhinolaryngology – Head and Neck Surgery, ASST Spedali Civili, University of Brescia, Brescia, Italy; ^10^ Division of Otolaryngology-Endocrine Head and Neck Surgery, Massachusetts Eye and Ear Infirmary, Harvard University, Boston, MA, United States; ^11^ University of Udine School of Medicine, Udine, Italy; ^12^ Department of Otolaryngology-Head and Neck Surgery, Galilee Medical Center, affiliated with Azrieli Faculty of Medicine, Bar-Ilan University, Safed, Israel; ^13^ Department of Endocrine Neoplasia and Hormonal Disorders, The University of Texas MD Anderson Cancer Center, Houston, TX, United States; ^14^ Head and Neck Service, Department of Surgery, Memorial Sloan-Kettering Cancer Center, New York, NY, United States; ^15^ Department of Hematology and Medical Oncology, Winship Cancer Institute, Emory University, Atlanta, GA, United States; ^16^ Department of Otolaryngology-Head and Neck Surgery, The Johns Hopkins University School of Medicine, Baltimore, Maryland, United States; ^17^ Division of Head and Neck Endocrine Surgery, Sarasota Memorial Health Care System, Sarasota, FL, United States; ^18^ Department of Pathology, University of Texas MD Anderson Cancer Center, Houston, TX, United States; ^19^ Department of Head & Neck Surgery, MD Anderson Cancer Center, Houston, TX, United States; ^20^ International Head and Neck Scientific Group, Padua, Italy

**Keywords:** thyroid neoplasms, fine needle aspiration, core needle biopsy, thyroid lymphoma, anaplastic thyroid cancer (ATC)

## Abstract

**Background:**

Both anaplastic thyroid carcinoma (ATC) and thyroid lymphoma (TL) clinically present as rapidly enlarging neck masses. Unfortunately, in this situation, like in any other thyroid swelling, a routine fine-needle aspiration (FNA) cytology is the first and only diagnostic test performed at the initial contact in the average thyroid practice. FNA, however, has a low sensitivity in diagnosing ATC and TL, and by the time the often “inconclusive” result is known, precious time has evolved, before going for core-needle biopsy (CNB) or incisional biopsy (IB) as the natural next diagnostic steps.

**Objectives:**

To determine the diagnostic value of CNB in the clinical setting of a rapidly enlarging thyroid mass, *via* a systematic review and meta-analysis of the available data on CNB reliability in the differential diagnosis of ATC and TL.

**Methods:**

A PubMed, Embase and Web of Science database search was performed on June 23th 2021. Population of interest comprised patients who underwent CNB for clinical or ultrasonographical suspicion of ATC or TL, patients with a final diagnosis of ATC or TL after CNB, or after IB following CNB.

**Results:**

From a total of 17 studies, 166 patients were included. One hundred and thirty-six were diagnosed as TL and 14 as ATC following CNB. CNB, with a sensitivity and positive predictive value of 94,3% and 100% for TL and 80,1% and 100% for ATC respectively, proved to be superior to FNA (reported sensitivity for TL of 48% and for ATC of 61%). Furthermore, the need for additional diagnostic surgery after CNB was only 6.2% for TL and 17.6% for ATC.

**Conclusions:**

Immediately performing CNB for a suspected diagnosis of ATC and TL in a rapidly enlarging thyroid mass is more appropriate and straightforward than a stepped diagnostic pathway using FNA first and awaiting the result before doing CNB.

## Introduction

Although of a low incidence, thyroid lymphoma (TL) and anaplastic thyroid carcinoma (ATC) are aggressive malignancies in need of urgent management. Presentation is similar (usually a 60-70-year-old female with a rapidly enlarging neck mass over weeks to months, and with symptoms like neck pain, dysphagia, hoarseness and stridor), but prognosis and management differ (1–6). Urgent management would benefit from an evidence-based strategy excluding unnecessary delays in reaching a precise diagnosis.

TL accounts for 0.4-5% of thyroid malignancies and for less than 2-3% of all extranodal lymphomas (3, 7–9). Most TLs are non-Hodgkin B-cell lymphomas. The most common subtype (>50%) is diffuse large B-cell lymphoma (DLBCL), followed by mucosa associated lymphoid tissue lymphoma (MALTL; about 10–30%) (3, 10). T-cell and Burkitt thyroid lymphomas are extremely rare (11, 12). Subtype classification is of great importance as prognosis and therapy depend on the lymphoma histotype. MALTL tends to be less aggressive than DLBCL and T-cell lymphomas. The latter two need more intensive chemotherapy or chemoradiotherapy (10). Overall, TL responds well to non-surgical treatment with a disease-specific survival of up to 79% (1). In contrast, ATC responds much less to treatment (5). While accounting for <2% of thyroid malignancies, ATC accounts for 25% of all thyroid cancer-related deaths (5, 13). In selected ATC, where R0, or at most R1 resection with acceptable morbidity seems likely, extensive surgery needs prompt adjuvant intensive radiotherapy and/or chemotherapy (14); when surgery is not an option, primary chemoradiotherapy, palliative radiation, systemic therapy, or best supportive care is offered. Prognosis is poor, with a median survival of 5 months and a 2-year overall survival of 10% (5, 13). Regardless of the treatment, most patients have a rapidly growing, extensively locally invading tumor, causing death with uncontrolled local disease and distant metastases. One series describes 36% of their patients dying of airway obstruction, the remainder succumbing to generalized disease progression (13, 15). Nonetheless, recent molecular-based personalized treatment strategies achieve better cure rates, with the combination of dabrafenib (inhibitor of BRAF) and trametinib (inhibitor of MEK) now being FDA (U.S. Food and Drug Administration)-approved in ATC patients with the BRAFV600E mutation (5, 16).

Imaging studies in the acute setting consist of ultrasound (US) as well as cross-sectional imaging. US typically shows a heterogeneous echogenicity, a diffuse infiltration into surrounding tissues, an irregular shape and an increased vascularity. Calcifications would be more in keeping with ATC, whereas the presence of echogenic strands and enhanced posterior echoes are more indicative of TL (3, 17). Cross-sectional imaging is essential to visualize the relationship of the tumor to the major vessels, trachea and esophagus, which guides surgical decision making. Computed tomography (CT) is preferred over magnetic resonance imaging (MRI): the shorter data acquisition time in patients with often some form of respiratory distress results in more accurate images. CT may show calcification, necrosis and heterogeneous attenuation in ATC; homogeneous attenuation without calcification or necrosis suggests TL (6). On MRI ATC shows also a moderate-to-marked heterogeneous enhancing with central non enhancing areas of necrosis, and mixed signal on T1- and T2-weighted images, whereas TL tends to be homogenous, similar to the CT aspect of TL (18).

Tissue analysis using US-guided cytology and/or histology is essential for establishing an accurate diagnosis. In this context, FNA, generally performed using a 21-25 Gauge needle on a 10 cc syringe under ultrasound guidance, with up to 8 needle passes, depending on the on-site judged appropriateness of the obtained material, is often the initial diagnostic tool (5, 19, 20). The real-world reported sensitivity of FNA is 48% for TL (21) and 50-61% for ATC (22, 23); for ATC an FNA-based correct diagnosis in >60% requires immunohistochemistry (IHC) on cell-blocks from cell-rich aspirates (24). These low figures are attributable to a high rate of false negative results, related to the pathogenesis of both malignancies. TL often develops in a background of Hashimoto’s thyroiditis (HT), chronic autoimmune stimulation being considered as the main trigger for malignant transformation (7, 25–27). The proportion of TL with a known history of HT is 80%, conversely, compared to the general population, patients with HT have a 60-fold increased risk of developing TL (28, 29). Distinguishing MALTL from HT is particularly difficult on cytology; both conditions may coincide and their lymphocytic populations show a similar morphology (30, 31). ATC may show extensive necrosis (32) and it causes little surprise that FNA often misses viable tumor.

Resort to US-guided core needle biopsy (CNB), generally performed under local anesthesia and ultrasound guidance, stabbing 2-3 times using a 16-20 Gauge spring-activated 2 cm excursion needle, comes currently after (persistently) inadequate results with FNA (5, 13, 20, 33). Nonetheless, Na et al. recommended immediate CNB in patients with thyroid masses with uncommon clinical and radiological features; the recommendation was based on a small amount of retrospective cohort studies, not specific for ATC and TL (34).

**Figure 1:**
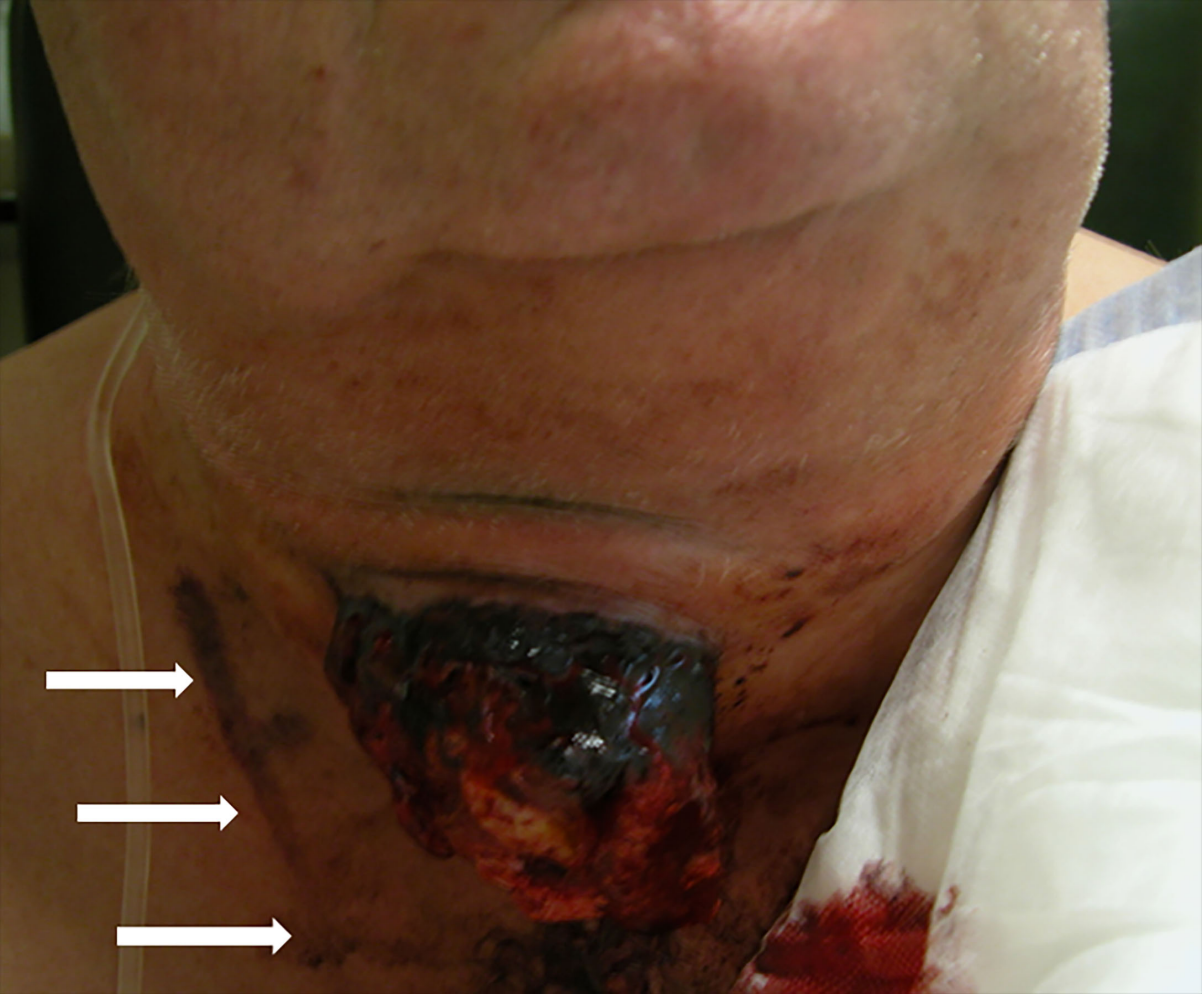
ATC, growing through the dehiscent incision of the previous biopsy, during the palliative radiotherapy. Note the tattoo on the skin of the patient demarcating the radiation field (arrows). Reproduced and modified with permission from Head and Neck Cancer Imaging. 2021:3rd Edition. R. Hermans (Editor); V. Vander Poorten: (I) Epidemiology, Risk Factors, Pathology, and Natural History of Head and Neck Neoplasms. Springer Nature Switzerland AG, Cham, Switzerland.

CNB aside, surgical incisional biopsy (IB; also referred to as “diagnostic surgery”; making a skin incision under local or general anesthesia to take a substantial tissue sample (2-3 cm³) of the tumor, avoiding the necrotic parts) would seem the obvious choice in overcoming the FNA inadequacies. Enough tissue of preserved architecture is thus obtained to effect routine histology and ancillary studies, including IHC for lymphoma subtyping and, defining molecular markers for ATC such as Ki-67, p53, *BRAF V600E* mutations and eventual *NTRK, ALK* or *RET* fusions (5, 24, 25, 35–38). However, IB in patients with TL or ATC carries significant risks. The procedure is invasive, time-consuming and involves hospital admission, general anesthesia and intubation of patients often already in respiratory distress. Extubation is unsure, whereas tracheotomy upon failed extubation is hazardous or even impossible, due to the compressive thyroid mass. Moreover, wound healing of the incision is often compromised (**Figure 1**).

Accordingly, to reduce the risks of IB and to minimize the delay to treatment, CNB seems a diagnostic tool more sensitive than FNA. Less invasive than IB, CNB allows for the same adequate IHC and molecular marker analysis. For diagnosing malignant lymphomas in the neck outside the thyroid, as well as for the more common thyroid neoplasms, CNB has yielded satisfactory results with reported sensitivities of up to 100% (35, 39, 40). In the narrower context of diagnosing ATC and TL, a thorough review of the reliability of CNB is lacking. This prompted the present systematic review and meta-analysis, to assess the value of CNB in diagnosing ATC and TL by calculating sensitivity, PPV, and the rate of diagnostic surgery.

## Materials and methods

### Search strategy and study selection

**Figure 2:**
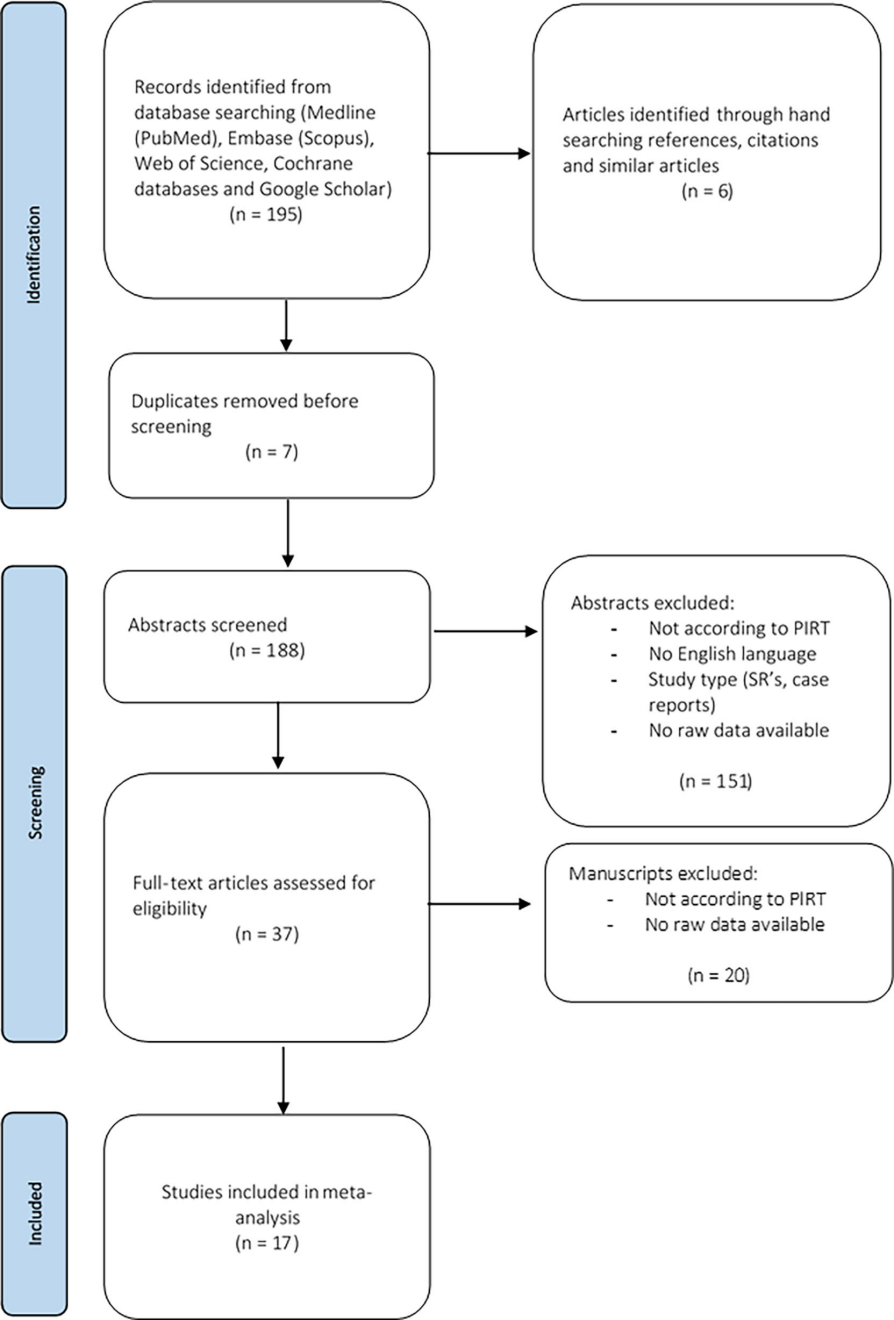
Flow diagram of the selection process of the relevant manuscripts, based on PRISMA guidelines.

The present systematic review was conducted according to the Preferred Reporting Items for Systematic Reviews and Meta-Analyses (PRISMA) guidelines (41). Manuscripts were selected if they complied with the following PIRT model (Population, Index test, Reference test, Target condition) (42): the population of interest (P) were patients who underwent CNB because of clinically or US features suggestive for ATC or TL, or patients with a final diagnosis of ATC or TL after CNB, or after IB following CNB; the index test (I) was CNB, the reference test (R) being the final diagnosis based on IB, or the surgical pathology or the clinical evolution. The target conditions (T) were ATC or TL, without further specification. Medline (PubMed), Embase (Scopus), Web of Science, and the Cochrane database were searched using the keywords “core-needle biopsy”, “thru-cut biopsy”, “anaplastic thyroid carcinoma”, “thyroid lymphoma”, “thyroid neoplasms”, and “thyroid nodules” on June 23^th^ 2021 (**Supplemental Figure S1**). Publications in English were included. Case reports, narrative and systematic reviews were excluded. Abstracts that did not meet the PIRT criteria were also excluded. The included abstracts were uploaded in a citation manager. Full texts were screened. Manuscripts not meeting the PIRT criteria were excluded. Only studies investigating the diagnostic value of CNB and providing raw data concerning the diagnostic value of CNB were included. Finally, hand-searching references, citations, and similar articles was undertaken. **Figure 2** shows the PRISMA diagram representing this selection process. A risk of bias analysis of the selected studies was performed using the QUADAS-2 tool (RevMan 5.4; **Supplemental Figure S2**) (43). Ethical exemption was provided by the Education-Support Committee (OBC) of the Research Ethics Committee of the KU Leuven.

### Data extraction

The following data were extracted : (1) general features of the study (author, year of publication, country, period of patient enrollment, study type, number of patients), (2) demographics (gender ratio, mean age and range), (3) patients’ characteristics, (4) number of patients with final diagnosis of ATC or TL, (5) number of patients with a rapidly growing neck mass who had undergone CNB, (6) overall results and complications of CNB, and (7) number of patients that underwent IB.

### Statistical analysis

For meta-analysis on diagnostic test accuracy, bivariate models that account for the relationship between specificity and sensitivity, producing a summary receiver operating characteristic curve, are preferred when diagnostic thresholds vary between studies. When the same diagnostic threshold is used across all included studies and there is little statistical heterogeneity, proportional meta-analysis can be applied for producing summary estimates of diagnostic accuracy measures such as sensitivity and specificity. The latter approach is used in this study, more so because information on specificity is lacking for most of the studies, precluding the option of a bivariate approach.

A meta-analysis is conducted to obtain - where possible - estimates of proportions quantifying sensitivity, specificity, PPV and NPV. The statistic I_2 was performed to assess heterogeneity. The analysis is based on a double arcsine-transformation (Freeman-Tukey transformation) of the proportions and has been performed using SAS software (version 9.4 of the SAS System for Windows).

The RevMan 5.4 program (Cochrane Training) was used to calculate and visualize effect measures of diagnostic value of CNB. Sensitivity and specificity were calculated for the studies included in the meta-analysis. Extracted data were eventually pooled to define a pooled sensitivity, positive predictive value (PPV), and proportion of IB. Negative predictive value (NPV) and specificity were calculated only for manuscripts including true negatives (44–46). CNB with a diagnosis of ATC or TL was classified as positive/concordant; it was classified as negative in all other situations (e.g. CNB with a diagnosis of thyroid cancer of a different origin, neoplastic lesion, benign lesion, insufficient material for a histologic analysis, inconclusive, or missed target). As defined in the literature, surgery was considered “diagnostic” if it had been the first way to obtain an accurate diagnosis (40).

## Results

### Studies and demography

A total of 17 studies (11 following systematic literature search and 6 after hand-searching) met the inclusion criteria for the present systematic review (11, 12, 23, 25, 30, 40, 44–54). The general features and demographic data are summarized in **Table 1** and **Supplementary Table 1**. All studies but one were retrospective (50).

**Table 1:** General characteristics of the included studies.

Reference (year)	TYPE	Country	Study period^*^	gender ratio (fem:male)	Mean age in years(range)	Number of patients ^°^
Alzouebi et al. (2012) (12)	TL	UK	1970-2010	4.4:1	66 (20-90)	70
Buxey et al. (2012) (55)	TL	Australia	1996-2009	N/A	72 (50-90)	7
Ha et al., 2016) (40)	TL and ATC	Korea	2000-2012	PTL 2.3:1 ATC 4.9:1	PTL: 59 (30-90) ATC: 67 (20-91)	40 59
Hahn et al. (2013) (45)	TL	Korea	2006-2010	N/A	N/A	10 †
Kakkar et al. (2019) (25)	TL	India	2009-2015	2.7:1	64,6 (40-76)	11
Jin et al. (2007) (54)	TL	Korea	2003-2005	2:1	62,5 (56-72)	6
Nam et al. (2012) (47)	TL	Korea	1995-2010	3.3:1	60,8 (43-82)	13
Pradhan et al. (2019) (48)	ATC	India	1991-2013	1.2:1	58 (36-80)	100
Quesada et al. (2016) (11)	TL	USA	2000-2015	0.8:1	41 (19-49)	7
Ruggiero et al. (2005) (49)	TL	USA	1977-2004	2.7:1	76,5	22
Sarinah et al. (2010) (50)	TL	Malaysia	1998-2006	1.8:1	58 (31-82)	17
Sharma et al. (2016) (30)	TL	USA	2000-2014	1:1	67 (N/A)	75
Stacchini et al. (2015) (46)	TL	Italy	2001-2013	1.2:1	66 (33-89)	13
Suh et al. (2013) (23)	ATC	Korea	2001-2011	2.6:1	70,5 (54-81)	18
Wu et al. (2016) (56)	TL	Taiwan	1992-2015	1.5:1	67,9 (54-83)	10
Xu et al. (2021) (44)	TL and ATC	China	2013-2018		N/A (39-75) ^‡^	24 ^‡^
Yang et al. (2015) (53)	TL	China	1995-2012	0.7:1	68,8 (N/A)	12
**Total, n**						**514**

N/A, not available.

^*^ Period of patient enrollment.

^°^ Number of all patients included in the study. Not equal to number of patients who underwent CNB.

† refers only to patients included in the study with atc or ptl, excluding patients with diagnosis other than atc or ptl.

^‡^ Refers to entire study population, including patients with diagnosis other than ATC or PTL. Data on gender ratio was therefore not incorporated in **Table 2**.

### Patient profile at presentation

**Table 2** summarizes the descriptive statistics on symptoms at presentation; 79% of patients with ATC and 88% with TL presented with a rapidly enlarging neck mass. A history of HT or concurrent HT was present in 31% of patients with TL but absent in patients with ATC.

**Table 2:** Patient characteristics at presentation^*^.

**Symptoms**	**ATC**	**TL**
Pooled gender ratio (female:male)	2:1 (117:60)	1.9:1 (211:109)
Rapidly enlarging neck mass (%)	79 (140/177)	88 (275/313)
Dysphagia (%)	40 (40/100)	31 (64/207)
Airway compression/dyspnea (%)	38 (38/100)	31 (47/151)
Hoarseness (%)	44 (44/100)	32 (46/143)
Stridor (%)	8 (8/100)	16 (15/92)
Hashimoto’s thyroiditis ^°^ (%)	0 (0/100)	31 (29/93)
Increased serum TSH (%)	10 (5/52)	50 (24/48)
Neck pain (%)	27 (27/100)	17 (16/92)

^*^ Symptoms not described in all studies. Ratios hold for patients included in studies that did report these symptoms.

^°^ Concurrent Hashimoto’s thyroiditis or previous history of Hashimoto’s thyroiditis.

### Qualitative analysis: Diagnostic role of CNB

The included studies differed as regards the role of CNB in diagnosing ATC or TL and thus were categorized in two groups. The first group applied CNB after inconclusive FNA; the second group applied CNB initially.

#### Application of CNB after FNA

Current guidelines advise CNB or IB after a non-diagnostic FNA (5, 13, 33). Repeat FNA still has a non-diagnostic rate of 20-38% (45), hence, several authors advised application of CNB after a single inconclusive FNA (25, 45, 50, 54). A combinative approach in which both FNA and CNB are initially performed was also proposed, on the basis that they complement each other, FNA being better suited for flow cytometry and CNB correlating better with histology (11). Stacchini et al. reported a 100% sensitivity and specificity in diagnosing TL when flow cytometry was applied to FNA, but emphasized that histological confirmation of diagnosis using CNB was necessary (46). Ruggiero et al., on the other hand, did not include CNB in their diagnostic pathway for TL, going immediately from FNA to IB in all patients (49).

#### Initial application of CNB

It seems overly expensive and time-consuming to sequentially perform FNA and CNB in all patients with suspected ATC or TL. Sharma et al. pointed out that CNB was delayed by an average of one week after a non-diagnostic FNA (30). As reported by Matsuzuka et al., patients died of progressive lymphomas before a definitive diagnosis could be made following a non-diagnostic FNA (57). At the same time, it has been suggested that CNB enables reducing the rate of IB in patients with ATC or TL, obviating surgical risk, morbidity, and unnecessary costs (40). Many authors support the initial application of CNB as the most appropriate initial diagnostic test when clinical signs and/or US features suggest TL or ATC, without firstly performing FNA (12, 30, 40, 44, 47, 48, 53, 55, 56).

### Complications of CNB

Most included studies reporting on CNB-related complications did not note any patient discomfort, bleeding, or tumor seeding (40, 44, 45, 54, 56), although the latter would admittedly be difficult to assess. Nam et al. reported some intraparenchymal hemorrhage that merely needed simple compression (47). Hematoma rate for CNB in thyroid pathology was recently reported to be 2.4% (58). Targeting lymphadenopathy, when present, can help reduce hematoma risk.

### Meta-analysis

**Figure 3:**
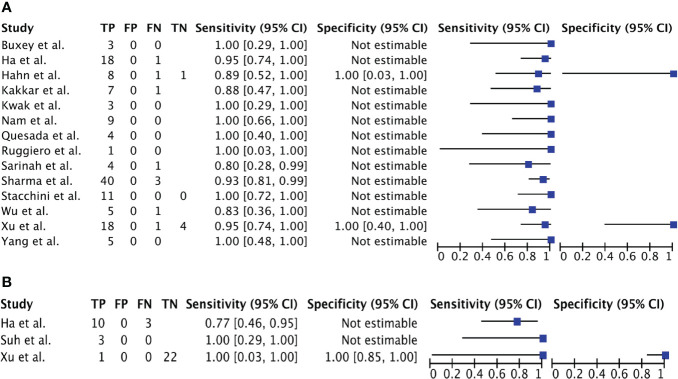
**(A)**. Forest Plot 1: meta-analysis on the role of CNB in PTL. **(B)**. Forest Plot 2: meta-analysis on the role of CNB in ATC.

Of the 17 studies included, 15 could be pooled in a meta-analysis; the remaining 2 did not provide adequate raw data (12, 48). Twelve studies included patients with TL (11, 25, 30, 45–47, 49, 50, 54–56, 59), one included only patients with ATC (23) and two both ATC and TL (40, 44). Raw data, sensitivity, and specificity - when possible - for each included study are shown in **Figures 3A, B**.

### Quantitative analysis: Diagnostic value of CNB and rate of IB

Pooling of extracted data from the 17 studies resulted in a total of 17 patients with established diagnosis of ATC, 14 of which were diagnosed with CNB; and a total of 145 patients with established diagnosis of TL, 136 of which were diagnosed with CNB (**Table 3**).

**Table 3:** Pooled data of all included studies.

Final diagnosis	CNB			
	ATC	TL	inconclusive	other
ATC	14	0	3	–
PTL	0	136	9	–
Other	–	–	–	4 ^*^

PTL, Thyroid lymphoma; ATC, anaplastic thyroid carcinoma; CNB, CORE NEEDLE BIOPSY.

^*^ Xu et al. (44): subacute thyroiditis (n=1), Hashimoto’s thyroiditis (n=1), RIEDEL thyroiditis (n=1). Hahn et al. (45) Hashimoto’s thyroiditis (n=1).

Meta-analysis methods rely on variability within and between studies. However, there was no within and no between variability in all eligible studies regarding PPV and specificity (so no summary statistics are estimated for these diagnostic measures): all studies reported a PPV and a specificity of 100%, both for ATC and TL.

Assessment of heterogeneity showed an I-2 statistic of -24.1 for sensitivity (p = 0.664; no heterogeneity detected) and of -103.4 for NPV (p=0.4832; no heterogeneity) so a fixed-effects meta-analysis could be performed.

Calculation of sensitivity of CNB and rate of IB for ATC and TL separately indicate the following. For diagnosing TL, CNB shows a sensitivity of 94,3% (90,6 – 97,1%) and a NPV of 68% (34-94%). CNB shows a slightly lower diagnostic value for diagnosing ATC with a sensitivity of 80,1% (59,4 – 94,6%). The proportion of patients needing IB is 6.2% for TL and 17.6% for ATC (**Table 4**).

**Table 4:** Results of the meta-analysis – pooled diagnostic performance measures.

CNB	ATC	TL
Sensitivity (%)	80.1	94.3
Specificity (%)*	100	100
Positive predictive value (%)*	100	100
Negative predictive value (%)	NE	68.0
Diagnostic surgery (%)	17.6	6.2

NE, not estimated.

* descriptive statistics.

## Discussion

For obtaining cells or tissues for microscopical examination in the situation of a patient presenting with a rapidly enlarging thyroid mass, FNA is the instinctive first test to be performed, but its limitations and low sensitivity have already been observed. In this context, the present meta-analysis suggests a superior diagnostic value of CNB, with a sensitivity and PPV of 94.3% and 100% for TL, 80.1% and 100% for ATC. The meta-analysis also suggests that CNB enables reducing the need for diagnostic surgery to 6.2% for patients with TL, to 17.6% for ATC and to 7.4% for both entities. In comparison, the need for IB after FNA is reportedly 34% for patients with ATC, 37.9% for TL, and 35.4% for both entities (40).

Besides more accurate typing a clear advantage of CNB over FNA is the more reliable IHC/genetic analysis. This is especially relevant since recent series using targeted therapy in *BRAF V600E*-mutant ATC have reported promising results, and a substantial clinical benefit (OS of 31,5% at 2 years) through dabrafenib and trametinib (37). In the absence of BRAF V600E mutation, *ALK*, *RET* and *NTRK* fusions, when present, can be targeted; checkpoint inhibitors have their place in case of high PD-L1 expression (5).

Risks of IB have been mentioned – hence this procedure is best reserved for the occasions where CNB is inconclusive. The main arguments of specialists who are reluctant to implement CNB are the requirement of a radiologist and imaging infrastructure on site, local anesthesia, a higher cost, more discomfort, risk for tumor seeding and a higher bleeding risk (45). However, looking critically at the literature, only self-limiting hemorrhages were reported, the incidence of post-CNB hematomas ranging between 0.02% and 2,4% (58, 60).

**Figure 4:**
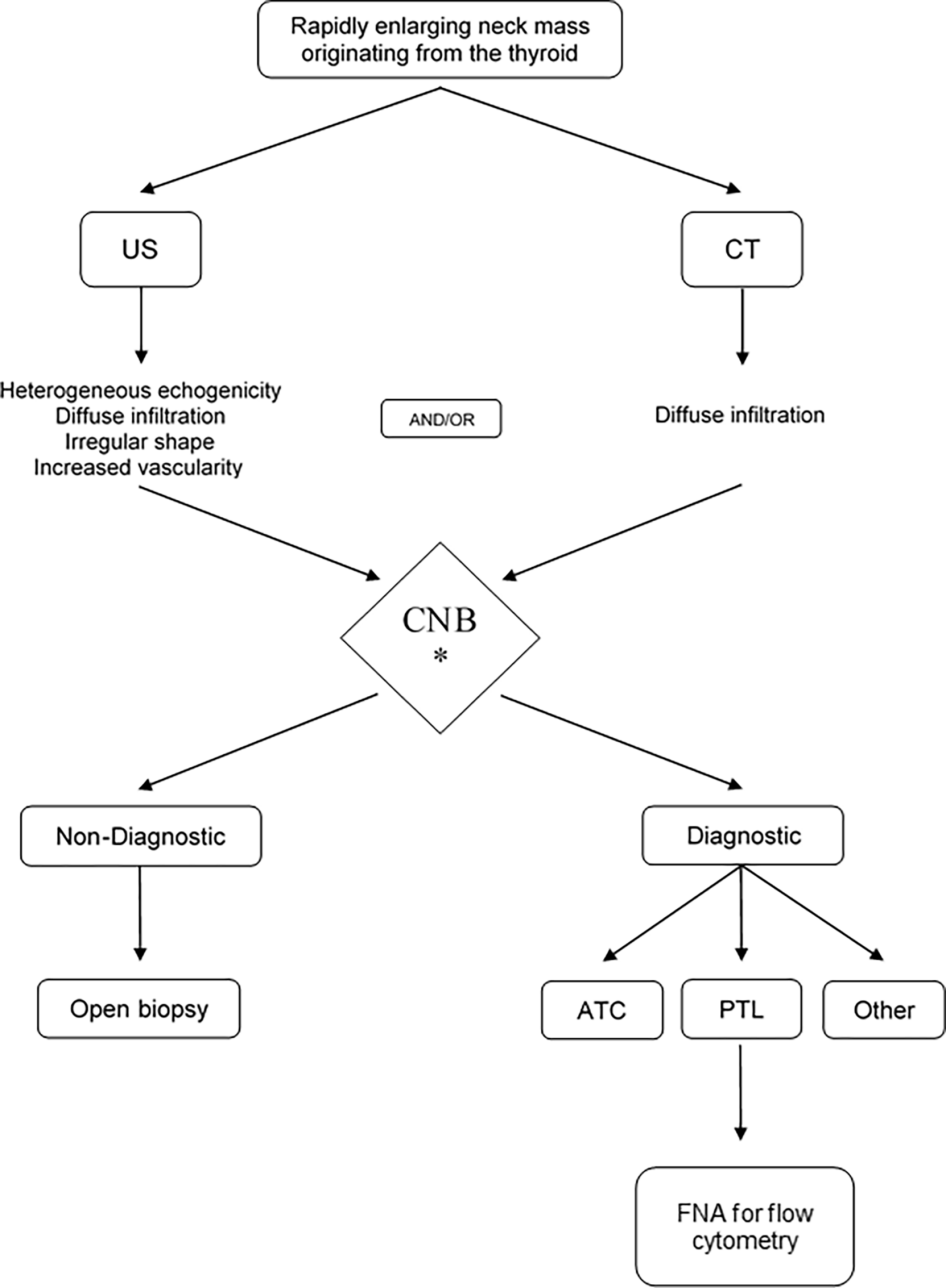
The proposed management algorithm for diagnosis of anaplastic thyroid carcinoma and thyroid lymphoma. US, ultrasound; CT, computed tomography; CNB, core needle biopsy; TL, thyroid lymphoma; ATC, anaplastic thyroid carcinoma.

The present first systematic review and meta-analysis assessing the diagnostic value of CNB in patients with ATC and TL indicates the high sensitivity and PPV of the procedure, the low need of diagnostic surgery and rare complications. It thus seems sensible to infer that CNB is the most appropriate initial diagnostic tool for obtaining cells or tissue for microscopical examination. Based on the flowchart suggested by Wu et al. (56) and the results of the present meta-analysis, an updated flowchart is now suggested (**Figure 4**). Recently in April 2022, ten months following the literature search of the current meta-analysis, an Italian prospective series compared FNA and CNB in the patient group presenting with a rapidly growing thyroid mass. The findings in the current meta-analysis were corroborated by this report on 13 TL and 33 ATC, worked-up in an experienced national tertiary referral center for thyroid cancer. In ATC, FNA was able to correctly identify histotype in only 3 patients (12.5%; PPV 17.1%) whereas CNB did so correctly in all 33 (PPV 100%). FNA correctly diagnosed TL in 7 of 13 patients (53,8%; PPV 61.5%), whereas CNB correctly diagnosed 12 of 13 TL (92%; PPV 92.3%) (20).

The obvious limitations to this systematic review and meta-analysis follow the inherent retrospective nature of the included retrospective studies dealing with two rare disease entities. Selection bias is difficult to eliminate. Ideally, studies should share the same inclusion criteria. First, however, the limited available literature necessitates allowance of studies with broader inclusion criteria than just TL or ATC, when they do offer the necessary raw data; these were then selectively extracted according to predefined inclusion criteria. Second, also of studies focusing on ATC and TL, inclusion criteria differed between the included manuscripts; most studies did not perform CNB primarily but only after inconclusive FNAs, and mostly CNB was performed in less patients than FNA; this selection bias may underestimate the diagnostic value of CNB, as evidenced in the study by Matrone et al. (20)

Also, following the inclusion criteria of many of the studies selecting only patients with TL and ATC, specificity and NPV could not be calculated for these patients, and the PPV of 100% should be interpreted with caution.

Finally, not all studies provide a detailed description of the CNB technique, nor of the experience of the radiologists and pathologists involved, which is likely to be quite divergent; these variables are thus not reflected in the findings of this meta-analysis.

## Conclusion

For patients presenting with a rapidly growing thyroid mass, CNB has a significantly higher sensitivity for in the diagnosis of ATC (80.1%) and TL (94.3%) than FNA (61% and 48%, respectively). Not associated with increased complications, and facilitating an earlier treatment, CNB thus seems the preferred initial step to obtain tissue diagnosis and start adequate treatment in this clinical scenario.

## Data availability statement

The original contributions presented in the study are included in the article/**Supplementary Material**. Further inquiries can be directed to the corresponding author.

## Author contributions

This paper was written by members and invitees of the International Head and Neck Scientific Group (http://www.IHNSG.com). VV, NG and AF contributed to conception and design of the study. VV and NG organized the database and performed the statistical analysis. VV, NG, and AT wrote the first draft of the manuscript. VV, NG, AT, MC, PC, OC, PG, OG-L, CP, GR, AR, OR, AS, ARS, YT, RT, MW, MZ and AF wrote the final draft of the manuscript. All authors contributed to the article and approved the submitted version.

## Funding

Supported by the Walter Vandeputte Head and Neck Cancer Fund (KU Leuven, Leuven, Belgium).

## Acknowledgments

The authors would like to sincerely thank Annouschka Laenen, PhD, from the Leuven Biostatistics and Statistical Bioinformatics Centre, KU Leuven, Leuven, Belgium, for the help in the statistical analysis.

## Conflict of interest

The authors declare that the research was conducted in the absence of any commercial or financial relationships that could be construed as a potential conflict of interest.

## Publisher’s note

All claims expressed in this article are solely those of the authors and do not necessarily represent those of their affiliated organizations, or those of the publisher, the editors and the reviewers. Any product that may be evaluated in this article, or claim that may be made by its manufacturer, is not guaranteed or endorsed by the publisher.
