# Supplementary figures and images for: Effectiveness of core needle biopsy in the diagnosis of thyroid lymphoma and anaplastic thyroid carcinoma: A systematic review and meta-analysis
Source: Front Endocrinol (Lausanne). 2022 Sep 20;13:971249. doi: 10.3389/fendo.2022.971249 (PMC9532007; doi:10.3389/fendo.2022.971249)

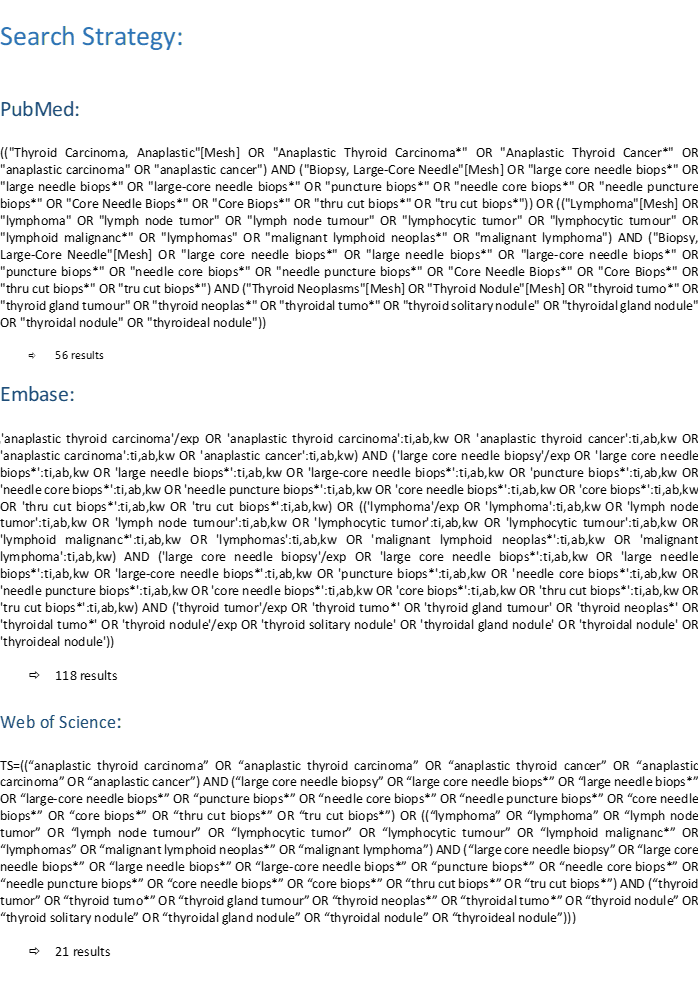

Supplement: Supplementary file 1 [file Image_1.tif]

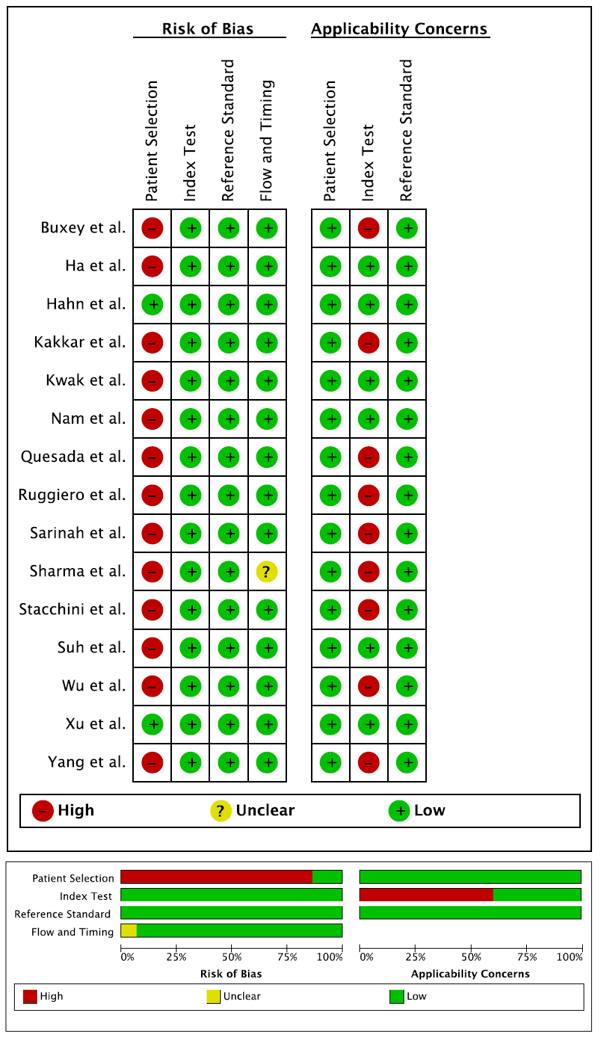

Supplement: Supplementary file 2 [file Image_2.tif]
