# Supplementary material for: Effectiveness of core needle biopsy in the diagnosis of thyroid lymphoma and anaplastic thyroid carcinoma: A systematic review and meta-analysis
Source: Front Endocrinol (Lausanne). 2022 Sep 20;13:971249. doi: 10.3389/fendo.2022.971249 (PMC9532007; doi:10.3389/fendo.2022.971249)
Supplement: Supplementary file 3 [file Table_1.docx]

**Supplementary Table 1.** **Study design of the included papers and way of patients’ enrolment**

| **Reference (year)** | **Study design** | **Patient enrollment** |
| --- | --- | --- |
| Alzouebi et al. (2012) [12] | Retrospective cohort study | Database screening of the Weston Park Hospital lymphoma database, to identify all patients diagnosed with TL between 1970 and 2010. |
| Buxey et al. (2012) [56] | Retrospective cohort study | Database screening of the Monash University Endocrine Surgery database, to identify all patients diagnosed with TL between 1996 and 2009. |
| Ha et al. 2016)[40] | Retrospective cohort study | Database screening of the Ajou University School of Medicine database, to identify all patients diagnosed with TL or ATC between 2000 and 2012. |
| Hahn et al. (2013)[45] | Retrospective cohort study | Database screening of the Samsung Medical Center, Sungkyunkwan University School of Medicine database, to identify all patients who underwent CNB after inconclusive FNA of the thyroid between 2006 and 2010. Patients who did not have ultrasonography-guided FNA prior to CNB or who underwent ultrasonography-guided FNA at another hospital were excluded. Patients were then divided into three groups based on ultrasonography features: Group A suspicious for papillary thyroid carcinoma, Group B suspicious for follicular (Hurthle cell) neoplasm, Group C suspicious for TL. Only data concerning patients in group C were extracted. |
| Kakkar et al. (2019)[25] | Retrospective cohort study | Database screening of the All India Institute of Medical Sciences, New Delhi database, to identify all patients diagnosed with TL between 2009 and 2015. |
| Jin et al. (2007)[54] | Retrospective cohort study | Database screening of the Ohio State University Wexner Medical Center and Virginia Commonwealth University database, to identify all patients diagnosed with ATC between 2000 and 2015. |
| Nam et al. (2012)[47] | Retrospective cohort study | Database screening of Samsung Medical Center, Sungkyunkwan University School of Medicine database, to identify all patients diagnosed with TL between 1995 and 2010. |
| Pradhan et al. (2019)[48] | Retrospective cohort study | Database screening of the Sanjay Gandhi Postgraduate Institute of Medical Sciences database, to identify all patients diagnosed with ATC between 1991 and 2013 |
| Quesada et al. (2016) (11) | Retrospective cohort study | Database screening of University of Texas MD Anderson Cancer Center database, to identify all patients diagnosed with aggressive B-cell lymphoma involving the thyroid gland between 2000 and 2015. |
| Ruggiero et al. (2005)[49] | Retrospective cohort study | Database screening of Milton Hershey Medical Center database, to identify all patients diagnosed with TL between 1977 and 2004. |
| Sarinah et al. (2010)[50] | Prospective cohort study | Consecutive enrollment of patients diagnosed with TL at the Department of Breast and Endocrine Surgery, Hospital Putrajaya, Malaysia, between 1998 and 2006. |
| Sharma et al. (2016)[30] | Retrospective cohort study | Database screening of the Mayo Clinic Lymphoma database, to identify all patients diagnosed with TL between 2000 and 2014. |
| Stacchini et al. (2015)[46] | Retrospective cohort study | Database screening of the Diagnostic Laboratory Department, Flow Cytometry Unit, Turin, to identify all patients who underwent thyroid FNA between 2001 and 2013. Only patients with final diagnosis of thyroid lymphoma and who underwent FNA with Flow Cytometry were reviewed. |
| Suh et al. (2013) [23] | Retrospective cohort study | Database screening of the Yonsei University College of Medicine database, Seoul, to identify all patients diagnosed with ATC between 2001 and 2011. |
| Wu et al. (2016)[ 57] | Retrospective cohort study | Database screening of the Tri-Service General Hospital database, Taipei, to identify all patients diagnosed with TL between 1992 and 2015. |
| Xu et al. (2021) [44] | Retrospective cohort study | Database screening of the Run Run Shaw Hospital database, Zhejiang University School of Medicine, to identify all patients presenting with sonographic features suspicious for TL between 2013 and 2018. |
| Yang et al. (2015)[53] | Retrospective cohort study | Database screening of the Chinese PLA General Hospital database, Beijing, to identify all patients diagnosed with TL between 1995 and 2012. |
| R = retrospective, P = prospective |  |  |
