# Supplementary material for: Effectiveness of core needle biopsy in the diagnosis of thyroid lymphoma and anaplastic thyroid carcinoma: A systematic review and meta-analysis
Source: Front Endocrinol (Lausanne). 2022 Sep 20;13:971249. doi: 10.3389/fendo.2022.971249 (PMC9532007; doi:10.3389/fendo.2022.971249)
Supplement: Supplementary file 4 [file Table_2.docx]

| DID THE PATIENT UNDERGO FNAC BEFORE CNB? | | | | | |
| --- | --- | --- | --- | --- | --- |
|  |  | YES | NO | UNKNOWN | NOTA |
| Buxey et al. (2012) [56] | TL | 3 | 0 | 0 |  |
| Ha et al. 2016)[40] | ATC+TL | 0 | 0 | 32 | study includes TL and ATC. No raw data available for for each pathology. 16/32 CNBs were performed after or together with FNAC, 16/32 CNBs were performed without FNAC |
| Hahn et al. (2013)[45] | TL | 10 | 0 | 0 |  |
| Kakkar et al. (2019)[25] | TL | 1 | 7 | 0 |  |
| Nam et al. (2012)[47] | TL | 3 | 6 | 0 |  |
| Quesada et al. (2016) (11) | TL | 0 | 0 | 4 | Available information: Four patients underwent core needle biopsy of the thyroidgland, and 3 patients underwent either total (n = 1) or partial(n = 2) thyroidectomy. Five patients underwent FNA beforeor concurrent with the biopsy. |
| Ruggiero et al. (2005)[49] | TL | 1 | 0 | 0 |  |
| Sarinah et al. (2010)[50] | TL | 0 | 0 | 5 |  |
| Sharma et al. (2016)[30] | TL | 40 | 3 | 0 |  |
| Stacchini et al. (2015)[46] | TL | 11 | 0 | 0 |  |
| Suh et al. (2013) [23] | ATC | 3 | 0 | 0 |  |
| Wu et al. (2016)[ 57] | TL | 5 | 1 | 0 |  |
| Xu et al. (2021) [44] | TL | 0 | 0 | 23 |  |
| Yang et al. (2015)[53] | TL | 0 | 0 | 5 |  |
| TOTAL |  | **YES** | **NO** | **UNKNOWN** |  |
| ATC  TL |  | 3  74 | 0  17 | 23  14  Ha et al.: 16 (unknown whether ATC or TL) | |
